# Target next-generation sequencing for the accurate diagnosis of *Parvimonas micra* lung abscess: a case series and literature review

**DOI:** 10.3389/fcimb.2024.1416884

**Published:** 2024-07-11

**Authors:** Dongmei Zhang, Boyang Fan, Yuan Yang, Chunguo Jiang, Li An, Xue Wang, Hangyong He

**Affiliations:** ^1^ Department of Respiratory and Critical Care Medicine, Beijing Institute of Respiratory Medicine and Beijing Chao-Yang Hospital, Capital Medical University, Beijing, China; ^2^ Department of Respiratory, Beijing Huairou Hospital, Beijing, China; ^3^ Department of Pulmonary and Critical Care Medicine, Center of Respiratory Medicine, China-Japan Friendship Hospital, National Center for Respiratory Medicine, National Clinical Research Center for Respiratory Diseases, Beijing, China

**Keywords:** *Parvimonas micra*, lung abscess, target next-generation sequencing, TNGS, diagnosis

## Abstract

**Background:**

*Parvimonas micra* (*P. micra*) has been identified as a pathogen capable of causing lung abscesses; however, its identification poses challenges due to the specialized culture conditions for anaerobic bacterial isolation. Only a few cases of lung abscesses caused by *P. micra* infection have been reported. Therefore, we describe the clinical characteristics of lung abscesses due to *P. micra* based on our case series.

**Methods:**

A retrospective analysis was conducted on eight patients who were diagnosed with lung abscesses attributed to *P. micra*. Detection of *P. micra* was accomplished through target next-generation sequencing (tNGS). A systematic search of the PubMed database using keywords “lung abscess” and “*Parvimonas micra/Peptostreptococcus micros*” was performed to review published literature pertaining to similar cases.

**Results:**

Among the eight patients reviewed, all exhibited poor oral hygiene, with four presenting with comorbid diabetes. Chest computed tomography (CT) showed high-density mass shadows with necrosis and small cavities in the middle. Bronchoscopic examination revealed purulent sputum and bronchial mucosal inflammation. Thick secretions obstructed the airway, leading to the poor drainage of pus, and the formation of local abscesses leading to irresponsive to antibiotic therapy, which finally protracted recovery time. *P. micra* was successfully identified in bronchoalveolar lavage fluid (BALF) samples from all eight patients using tNGS; in contrast, sputum and BALF bacterial cultures yielded negative results, with *P. micra* cultured from only one empyema sample. Following appropriate antibiotic therapy, seven patients recovered. In previously documented cases, favorable outcomes were observed in 77.8% of individuals treated with antibiotics and 22.2% were cured after surgical interventions for *P. micra* lung abscesses.

**Conclusions:**

This study enriches our understanding of the clinical characteristics associated with lung abscesses attributed to *P. micra*. Importantly, tNGS has emerged as a rapid and effective diagnostic test in scenarios where traditional sputum cultures are negative. Encouragingly, patients with lung abscesses caused by *P*. *micra* infection exhibit a favorable prognosis with effective airway clearance and judicious anti-infective management.

## Introduction

Primary lung abscesses typically result from infections leading to necrosis and cavitation of the lung tissue. Aspiration of oral-derived anaerobic bacteria represents the predominant etiology of primary lung abscesses (Bansal et al., 2013). Individuals with periodontal disease are at an elevated risk of developing anaerobic lung abscesses. Since anaerobic organisms necessitate specific culture conditions, conventional sputum culture often yields negative results in the detection of anaerobic bacteria. Anaerobic organisms were isolated only in a limited number of lung abscess cases (Yazbeck et al., 2014).

*Parvimonas micra* (*P. micra*) is a gram-positive anaerobic coccus prevalent in the oral cavity, particularly in the gingival crevices. *P. micra* has been associated with aspiration pneumonia (Yu et al., 2021), lung abscesses (Zhang et al., 2021), and empyema (Vilcarromero et al., 2023). Nevertheless, clinical insights into *P. micra-*related lung abscesses remain rare.

Given the challenges in culturing and identifying anaerobic bacteria due to their vulnerability to atmospheric exposure, innovative approaches utilizing next-generation sequencing of metagenomes (NGS) have shown promise in enhancing the positive detection rate of anaerobic bacteria (Zhang et al., 2021).

In this study, we present findings from eight cases of lung abscess caused by *P. micra*, with the pathogenic bacterium identified through genomic sequencing. Additionally, a systematic review of prior cases detailed in the published literature was conducted to delineate the clinical features of lung abbesses attributed to this pathogen, which may be unfamiliar to pulmonary healthcare providers.

## Patients and methods

### Patients

A retrospective analysis was conducted on patients who were diagnosed with lung abscesses and admitted to Beijing Chao-Yang Hospital between July 2022 and December 2023. The results from bronchoalveolar lavage fluid (BALF) pathogen testing using target next-generation sequencing (tNGS) indicated *P. Micra* as the predominant bacterium, consistent with the clinical presentation. Two physicians independently confirmed the association of lung abscess with *P. micra.* Comprehensive patient medical records, laboratory tests, examinations, and treatment modalities were compiled for review.

### tNGS testing

Bronchoscopies were performed within 5 days of hospital admission, with BALF samples obtained from the segmental bronchus corresponding to the identified lesions. Immediately following collection, 5 ml of BALF was aseptically preserved at −20°C and transported to Beijing KingMed Diagnostics Laboratory within 4 h. Cellular material within the samples was enriched, lysed, and subjected to DNA extraction from 500 μl of fluid as per established protocols. Subsequent to polymerase chain reaction amplification and purification, sequencing was performed utilizing a genetic sequencing platform (KM MiniSeqDx-CN) and an automatic nucleic acid-protein analyzer (Qsep100, BiOptic, Taiwan). Sequence data interpretation was facilitated by the Pathogenic Microbial Data Analysis and Management System 1.0 (KingMed Diagnostics, Co., Ltd., China), which comprises a bacterial minimal genome database consisting of 198 respiratory pathogens.

### Literature review

A systematic search of the PubMed database was conducted to identify journal articles, employing the search terms “lung abscess” and “*Parvimonas micra/Peptostreptococcus micros*”. The inclusion criteria included papers published from 01/01/1980, to 31/12/2023. Five articles documenting pulmonary abscesses associated with *P. micra*, with a collective total of nine patients, were contained in the review.

## Results

### Characteristics

The general characteristics of the eight patients are outlined in **Table 1**. Their ages ranged from 28 to 83 years, with four patients having diabetes and none presenting HIV infection or receiving immunosuppressive treatment. The median duration from symptom onset to definitive diagnosis was approximately 40 days. Notably, all eight patients exhibited poor oral hygiene, with manifestations including periodontal disease and dental disease.

**Table 1:** Clinical information of patients with *P. micra* lung abscess.

Case	Age	Gender	BMI	Underlying disease	Smoking/drinking	Oral hygiene	Symptoms	Time*	WBC 10^9^/L	NEU 10^9^/L	Hgb g/L	PCT ng/ml	CRP mg/dl
1	72	M	20.28	DM	N/N	Periodontal disease, dental calculus, false tooth	Cough sputum,	30 days	8.57	6.1	116	<0.05	4.06
2	67	F	21.30	–	N/N	dental calculus, teeth loss	Fever, cough, and sputum	50 days	5.3	3.41	117	<0.05	0.34
3	83	M	27.22	DM, HPT	Y/Y	Dental calculus, decayed teeth, teeth loss	Fever, cough and sputum, sore throat	2 months	10.56	7.6	109	0.06	11.5
4	54	F	24.43	–	N/N	Decayed tooth	Fever, cough, and sputum	2 months	6.99	4.85	94	<0.05	0.39
5	49	M	27.68	DM, HPT	Y/N	Abscess tooth, dental calculus	Fever, cough and sputum, chest pain	15 days	16.61	13.86	116	0.34	35.8
6	57	F	26.03	DM, HPT, CVD	N/N	Periodontal disease, false tooth, tooth loss	Fever, cough, and sputum	20 days	15.23	13.38	107	0.39	12.7
7	59	M	24.2	–	N/Y	Periodontal disease, dental calculus	Cough and sputum, chest pain, hemoptysis	70 days	6.56	4.32	127	<0.05	5.55
8	28	M	25.25	–	N/Y	Decayed tooth	Cough, dyspnea	14 days	17.61	15.54	106	0.46	4.21

M, male; F, female; DM, diabetes mellitus; HPT, hypertension; CVD, cerebrovascular disease; N, no; Y, yes.

*Time from clinical onset to diagnosis.

Primary symptoms were non-specific, yet common manifestations included fever (n = 5), cough (n = 8), and sputum production (n = 7). Additional complaints included sore throat, dyspnea, chest pain, and hemoptysis.

### Laboratory tests, chest CT, and bronchoscopy

Upon admission, routine blood tests revealed a median white blood cell count of 9.57 × 10^9^/L, with half of the patients exhibiting normal procalcitonin (PCT) levels.

**Figure 1:**
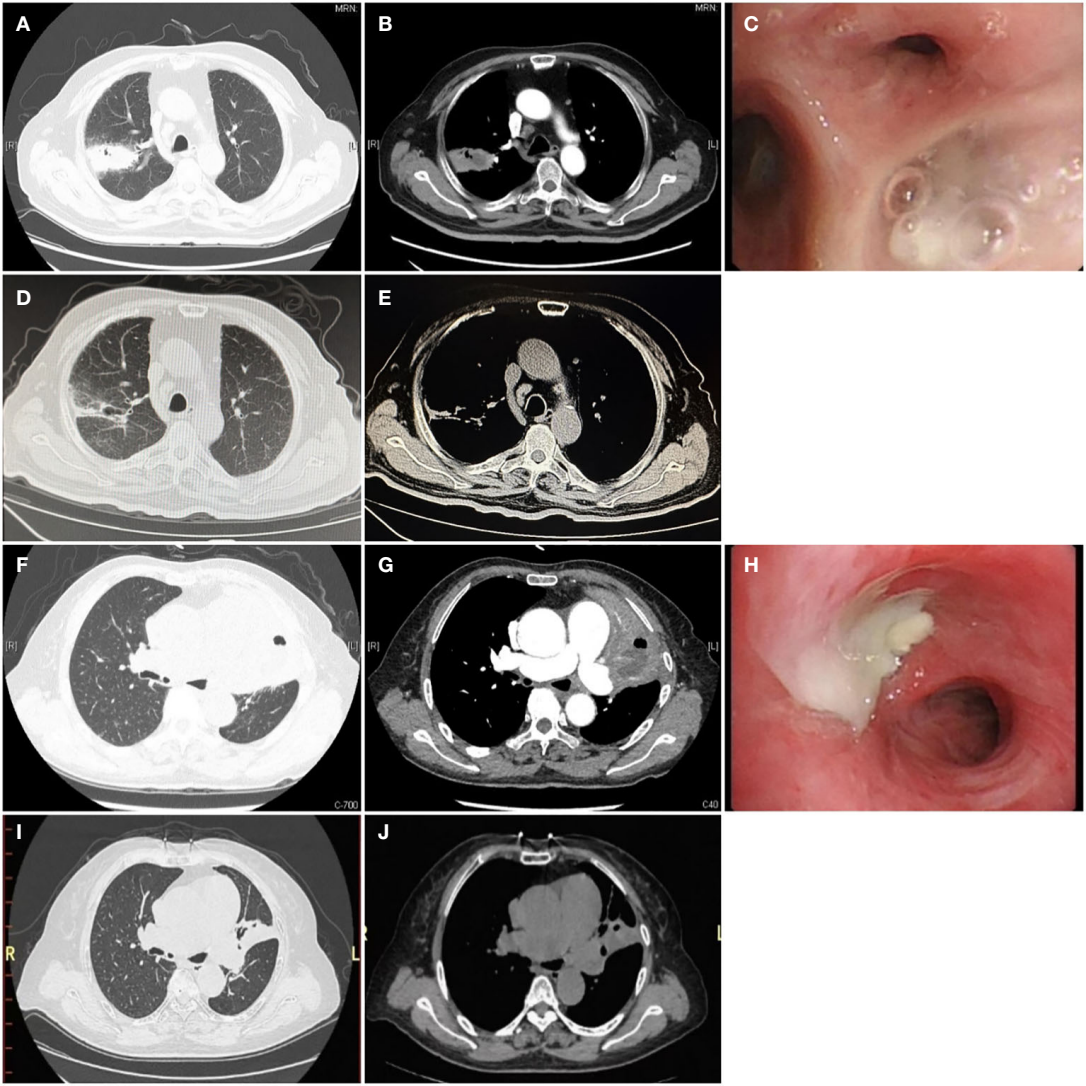
Chest computed tomography and bronchoscopy of *P. micra* lung abscess Computed tomography is displayed in the lung window **(A, D, F, I)**, soft tissue window **(B, E, G, J)**, and bronchoscopy **(C, H)**. **(A, B, F, G)** The initial CT scan showed large consolidations with necrosis and small cavities (white →) without a clear air-liquid level, which can be described as a “bubble levitation sign”. **(C, H)** The bronchial segment was obstructed by purulent secretions (→). **(D, E, I, J)** After treatment for 3 months, the follow-up chest CT showed significant absorption of the lesions.

Chest CT findings demonstrated large-scale consolidation with necrosis and small cavities (**Figure 1**), devoid of classical air-fluid levels. The boundaries of the focusing area were unclear and blurred. The lesions were located in the right upper lobe (n = 2), left upper lobe (n = 2), right middle lobe (n = 1), and inferior lobe of the right lung (n = 3). One patient was complicated with liver abscesses and empyema.

Bronchoscopy revealed purulent sputum (**Figure 1**) and inflammatory changes in the bronchial mucosa at the affected sites.

CT-guided pulmonary biopsy was performed on three patients to differentiate from lung cancer. Pathology indicated inflammation of the lung tissue.

### Microbiological examinations

Although gram-positive cocci were identified in five cases via sputum or BALF smears, bacterial cultures yielded negative results for all eight patients. However, tNGS analysis of BALF samples confirmed *P. micra* as the predominant bacterium. Anaerobic culture of the pleural effusion identified *P. micra* in one patient complicated with concomitant empyema. Additionally, tNGS reports indicated the presence of other bacteria including *Fusbacterium nucleatum* (n = 5), *Klebsiella pneumoniae* (n = 2), *Escherichia coli* (n = 1), *Streptococcus pneumoniae* (n = 1), and *Acinetobacter baumannii* (n = 1).

### Treatment and outcomes

Following diagnosis, patients received tailored anti-infection therapies such as piperacillin–tazobactam (n = 3), ampicillin–sulbactam (n = 2), moxifloxacin (n = 1), meropenem (n = 1), and imipenem–cilastatin (n = 1). Mucolytic drugs and airway clearance techniques were also employed to facilitate sputum expectoration.

Their symptoms improved, and chest CT scans indicated resolution of the lung abscesses. Subsequent to initial therapy, oral antibiotics were administered for 2 to 5 months. Seven patients achieved satisfactory outcomes, with almost no symptoms and almost completely absorbed lesions on chest CT. Two months later, one patient developed massive hemoptysis attributed to secondary *Enterococcus faecium* infection, leading to fatal pneumonia and sepsis.

### Literature review

A review of the literature identified other nine reported cases (**Table 2**), revealing cough (77.8%) and expectoration (77.8%) as the most common symptoms. Additional data indicated smoking history, alcohol consumption, periodontal conditions, and dental issues. The median time taken from clinical onset to make a diagnosis was 2 months. CT scan showed irregular mass shadows which had no obvious improvement after short-term initial empirical treatment. It is difficult to distinguish from malignant lesions. Thus, CT-guided percutaneous lung biopsies were performed in 55.6% of these patients. In published cases, metagenomics next-generation sequencing (mNGS) facilitated pathogen identification in more than half of the cases. The details of coinfections and treatment are shown in **Table 3**. A majority of *P. micra* lung abscess cases reported in the literature also displayed favorable outcomes, with 77.8% of patients cured via medication and 22.2% benefitting from surgical intervention.

**Table 2:** Clinical characteristics of *P. micra* in case series and in the literature.

Characteristic		Our case series (n = 8)	Literature cases (n = 9)
Age	Year median (range)	55.5 (28-82)	61.5 (46-82)
Gender	Male/female	5/3	8/1
Time from clinical onset to diagnosis (days)		40 (14-70)	60 (4-260)
Symptom No. (%)	Fever	5 (62.5)	3 (33.3)
	Cough	8 (100)	7 (77.8)
	Expectoration	7 (87.5)	7 (77.8)
	Chest pain	2 (25)	3 (33.3)
	Dyspnea	1 (12.5)	2 (22.2)
	Hemoptysis	1 (12.5)	3 (33.3)
	Sore throat	1 (12.5)	0
	Circulatory failure	0	1 (11.1)
Method pathogen detection	BALF NGS	8 (100)	3 (33.3)
	Lung tissue NGS	0	2 (22.2)
	BALF culture	0	1 (11.1)
	Blood culture	0	2 (22.2)
	Abscess culture	1 (12.5)	1 (11.1)
Underlying health status	Diabetes	4 (50)	1 (11.1)
	Hypertension	3 (37.5)	3 (33.3)
	Cerebrovascular disease	1 (12.5)	0
	Bipolar disorder	0	1 (11.1)
	Atrial fibrillation	0	1 (11.1)
	Heart valve replacement	0	1 (11.1)
	Healthy	4 (50)	3 (33.3)
Location of infection	Right upper lung lobe	2 (25)	3 (33.3)
	Right middle lung lobe	1 (12.5)	2 (22.2)
	Right lower lung lobe	3 (37.5)	0
	Left upper lung lobe	2 (25)	3 (33.3)
	Left lower lung lobe	0	2 (22.2)
Bronchoscopy		8 (100)	7 (77.8)
CT-guided percutaneous lung puncture		3 (37.5)	5 (55.6)
Prognosis	Cured with medicines	7 (87.5)	7 (77.8)
	Surgical resection	0	2 (22.2)
	All-cause mortality	1 (12.5)	0

BALF, bronchoalveolar lavage fluid; NGS, next-generation sequencing.

**Table 3:** Diagnosis, treatment, and prognosis of patients with *P. micra* lung abscess.

Case	Age	Gender	Lesion site	Biopsy method	Diagnostic method	Coinfection	Treatment	Prognosis
1	72	M	Right middle lung lobe	N	BALF tNGS	*K. pneumoniae*	Ampicillin sulbactam for 10 days, and amoxicillin clavulanate 500/125 mg every 8 h for 3 months	Cured with medicines
2	67	F	Left upper lung lobe	N	BALF tNGS	N	Meropenem 1.0 g every 8 h for 11 days, piperacillin tazobactam 4.5 g every 8 h for 14 days, moxifloxacin 400 mg once a day for 4 months	Cured with medicines
3	83	M	Right upper lung lobe	CT-GPLP	BALF tNGS	*F. nucleatum*	Meropenem 1.0 g every 12 h for 8 days, piperacillin tazobactam 4.5 g every 8 h for 6 days, moxifloxacin 400 mg once a day for over 5 months	Cured with medicines
4	54	F	Right lower lung lobe	CT-GPLP	BALF tNGS Lung tissue NGS	*A. baumannii*	Piperacillin tazobactam 4.5 g every 8 h for 15 days, moxifloxacin 400 mg once a day for 15 days, amoxicillin and metronidazole for 4 months	Cured with medicines
5	49	M	Right lower lung lobe	N	BALF tNGS	*P. endodontalis*	Ampicillin intravenous for 12 days and amoxicillin clavulanate for 2.5 months	Cured with medicines
6	57	F	Left upper lung lobe	CT-GPLP EBUS-TBNA	BALF tNGS	N	Ceftazidime 2.0 g every 8 h for 17 days, moxifloxacin 400 mg once a day for 5 months	Cured with medicines
7	59	M	Right upper lung lobe	TBLB	BALF tNGS	*P. endodontalis* *F. nucleatum*	Meropenem 1.0 g every 8 h for 17 days, moxifloxacin 400 mg once a day for 1.5 months, Imipenem cilastatin 1.0 g every 8 h for 8 days.	Death from *Enterococcus fecal sepsis*
8	28	M	Right lower lung lobe	N	BALF tNGS, culture of pleural effusion	*F. nucleatum*	Meropenem 1.0 g every 8 h for 12 days, Piperacillin tazobactam 4.5 g every 8 h for 20 days, moxifloxacin for 3 months	Cured with medicines
9 (Fukushima et al., 2023)	57	F	Right upper lung lobe	Surgical resection	Blood culture	N	Ceftriaxone, vancomycin, and azithromycin for 10 days	Cured with resection
10 (Yang and Su, 2021)	49	M	Left lower lung lobe	N	Blood culture	*A. odontolyticus*	Ceftriaxone and clindamycin Ampicillin sulbactam Amoxicillin/clavulanate 6 months	Cured with medicines
11 (Zhang et al., 2021)	61	M	Left upper lung lobe	CT-GPLP	Lung tissue NGS	N	Metronidazole 200 mg every 8 h for 3 months	Cured with medicines
12 (Zhang et al., 2021)	81	M	Right middle lung lobe	EBUS-TBNA	BALF NGS	*F. nucleatum*	Amoxicillin clavulanate 375 mg every 8 h for 2 months	Cured with medicines
13 (Zhang et al., 2021)	46	M	Left lower lung lobe	CT-GPLP	BALF NGS	*F. nucleatum*, *S. constellatus*	Moxifloxacin 400 mg once a day for 4 months	Cured with medicines
14 (Zhang et al., 2021)	82	M	Right upper lung lobe	CT-GPLP	Lung tissue NGS	*P. intermedia*, *S. constellatus*	Metronidazole 200 mg every 8 h for 5 months	Cured with medicines
15 (Zhang et al., 2021)	62	M	Left upper lung lobe	CT-GPLP	BALF NGS	*F. nucleatum*	Amoxicillin–clavulanate 375 mg every 8 h for 4 months	Cured with resection
16 (Yun et al., 2019)	62	M	Left upper lobe	N	BALF culture	N	Piperacillin–tazobactam 4.5 g every 6 h for 7 days, amoxicillin–clavulanic 875/125mg every 12 h for several weeks	Cured with medicines
17 (Gorospe et al., 2014)	67	M	Right upper lobe and middle lobe	CT-GPLP	Chest wall abscess culture	N	Levofloxacin for 7 days Clindamycin 600 mg every 8 h for 8 weeks	Cured with medicines

CT-GPLP, computed tomography-guided percutaneous lung puncture; EBUS-TBNA, endobronchial ultrasound-guided transbronchial needle aspiration; BALF, bronchoalveolar lavage fluid; NGS, next generation sequencing. N, no; M, male; F, female

## Discussion

The majority of lung abscesses are believed to be caused by the aspiration of anaerobic bacteria from the oral cavity, particularly from gingival crevices. *P. micra* is one of the most prevalent anaerobic microorganisms in the human oral cavity (Badri et al., 2019). However, due to challenges in laboratory culture, there are limited published data on the clinical characteristics of *P. micra* lung abscess, mainly in the last 5 years (Yun et al., 2019; Yang and Su, 2021; Zhang et al., 2021; Fukushima et al., 2023; Zhijun et al., 2023).

Anaerobic lung infections are often associated with poor oral hygiene conditions and inadequate airway protection (Hata et al., 2020). Zhang et al (Zhang et al., 2021). reported that patients with a long smoking history and poor oral hygiene are susceptible to *P. micra* lung abscesses. However, in our study, smoking was not identified as a significant risk factor, as only two male patients had a long history of smoking, whereas the other patients were non-smokers. Lai et al. reported four cases of childhood pneumonia and abscesses caused by oral obligate anaerobes, primarily *P. micra*, where poor oral hygiene was a crucial risk factor (Zhijun et al., 2023). Therefore, poor oral health is an important risk factor for *P. micra*–induced lung abscesses. In addition, alcoholism should also be noted as a common predisposing condition for aspiration (Kuhajda et al., 2015). Among our patients, three male patients had a history of regular alcohol consumption.

The symptoms observed in these patients were non-specific. All patients presented with a productive cough with sputum, and most experienced fever. Radiologically, chest CT scans revealed mass of lung consolidation with liquefactive necrosis and small cavities within the consolidation, without a distinct liquid–gas plane. Given the presence of small cavities with thick walls of consolidations on lung CT scan, along with non-specific symptoms, many patients underwent lung puncture biopsy for the differentiation of lung cancer. The lung biopsy pathology from these lung puncture biopsies revealed chronic inflammation of the lung tissue (Zhang et al., 2021). Gorospe et al. reported a case of a chest wall abscess of *P. micra* following CT-guided needle lung biopsy of the right lung consolidation (Gorospe et al., 2014).

In our case series, bronchoscopy revealed sticky secretion plugging in the bronchi as a feature of *P. micra* infection. For patients with lung consolidation and necrosis, bronchoscopy could be performed initially for pathogenic analysis. When empyema secretion plugging in the bronchial region was detected, without neoplasm, *P. micra* was isolated, and symptoms and radiological findings improved following targeted antibiotic therapy, percutaneous lung biopsy could be avoided.

The mNGS markedly improves pathogenic diagnosis, but it is expensive and involves procedural DNA and RNA sequencing, respectively. Target NGS is a more economical technology with a cost of approximately one-fifth of that of mNGS, with the similar advantages of rapid speed and high accuracy. The target detection of 198 pathogens include 80 bacteria, 79 DNA and RNA viruses, 32 fungi, and 7 mycoplasmas and chlamydia, which account for 95% respiratory infections (Li et al., 2022). Clinical data have shown that tNGS is effective and economical for diagnosis of respiratory infection (Li et al., 2022). However, NGS technology cannot achieve antibiotic sensitivity of the pathogen, and anaerobic culture remains important, particularly for determining antibiotic resistance. Additionally, as NGS often detects multiple bacterial species, clinicians must carefully ascertain the true pathogenic bacteria, by integrating patients’ symptoms, laboratory tests, and radiological findings. Compared with traditional culture, tNGS had a shorter turnaround time for positive pathogen detection (1 day vs. 4 days). Finally, for detected pathogens with high NGS sequencing read numbers, especially in RNA sequencing tests, this approach could be helpful for the differentiation of colonization and infection.

tNGS is only a method for detecting microbes rather than a diagnostic method. However, tNGS is designed to target highly suspected microbes when they are commonly recognized as pathogens that cause lower respiratory tract infections. Furthermore, after its routine application in the clinical diagnostic process in clinical practice for pathogen identification in our hospital, its role as an examination for diagnostic purposes becomes increasingly important. Therefore, we could have a positive view of its promising diagnostic usage in the future. Finally, the results from tNGS could not be held as the only diagnostic evidence. To establish a precise diagnosis for an infection, tNGS should be combined with other clinical information such as medical history, symptoms, laboratory, and radiological findings.

In bronchoscopy, if bronchial blockage by purulent secretions is observed, which leads to local hypoxia and creates conditions for anaerobic bacterial growth, promoting sputum excretion and maintaining airway patency are critical treatment procedures on the base of drug therapy. Chest physiotherapy by respiratory therapists, including the use of devices such as the Acapella valve and high-frequency chest wall oscillation devices, are helpful in clearing airway secretions (Polverino et al., 2017)

*P. micra* is susceptible to many antibiotics, including penicillin G, ampicillin, cefepime, vancomycin, and metronidazole (Boattini et al., 2018; Fukushima et al., 2023). In the case of lung abscess, intravenous antibiotics were administered for 2 to 4 weeks, followed by oral antibiotic treatment for 2 to 3 months until the chest radiographic lesions resolved. Proper drainage helps with absorption and hastens recovery. Although lung abscesses associated with *P. micra* have rarely been reported, it has a benign prognosis, as most patients recover after antibiotic treatment.

In patients who do not respond to antibiotic therapy, catheter drainage or surgical resection should still be considered (Herth et al., 2005). Both percutaneous tube drainage (Lee et al., 2022) and endoscopic drainage (Unterman et al., 2017) have been shown to effectively reduce abscess size and improve clinical outcomes. However, catheter placement in *P. micra-*related lung abscesses with small cavities can be challenging (Herth et al., 2005). Surgical resection may be necessary if antibiotic therapy is ineffective or in cases of life-threatening hemoptysis. In cases reported previously, two patients were cured after surgery.

This study has several limitations. First, this study has a small sample size, which may lead to bias. Furthermore, sputum or BALF cultures were negative for all patients. Finally, tNGS results do not cover all pathogens, which may be inaccurate in finding rare coinfected pathogens. To avoid missing important pathogens, retesting or conversion to mNGS would be considered, if the tNGS results not align with the clinical situation or if patients exhibited a poor response to treatment.

## Conclusion

In conclusion, our study systemically reports the characteristics of *P. micra*–related lung abscesses in adults based on the largest number of cases to date. Imaging features included mass consolidation with necrosis without a clear liquid–gas plane on chest CT, and sticky secretion plugging in the bronchial region on bronchoscopy. tNGS is an effective and cost-efficient tool for rapidly detecting pathogens. The lung abscesses caused by *P. micra* have a good prognosis with appropriate treatment. Improving oral health, promoting sputum excretion, and following an appropriate extended course of antibiotic treatment are crucial for recovery from *P. micra*-induced anaerobic lung abscesses.

## Data availability statement

The datasets presented in this study can be found in online repositories. The names of the repository/repositories and accession number(s) can be found in the article/supplementary material.

## Ethics statement

The studies involving humans were approved by Ethics Review Committee of the Beijing Chao-Yang Hospital, Capital Medical University. The studies were conducted in accordance with the local legislation and institutional requirements. Written informed consent for participation in this study was provided by the participants’ legal guardians/next of kin. Written informed consent was obtained from the individual(s) for the publication of any potentially identifiable images or data included in this article.

## Author contributions

DZ: Validation, Writing – original draft, Conceptualization. BF: Data curation, Investigation, Writing – original draft. YY: Data curation, Investigation, Writing – original draft. CJ: Data curation, Investigation, Writing – review & editing. LA: Conceptualization, Supervision, Writing – review & editing. XW: Data curation, Investigation, Writing – review & editing. HH: Project administration, Supervision, Visualization, Writing – review & editing.
